# The Effect of Viscous Air Damping on an Optically Actuated Multilayer MoS_2_ Nanomechanical Resonator Using Fabry-Perot Interference

**DOI:** 10.3390/nano6090162

**Published:** 2016-09-05

**Authors:** Yumei She, Cheng Li, Tian Lan, Xiaobin Peng, Qianwen Liu, Shangchun Fan

**Affiliations:** 1 School of Instrumentation Science and Opto-electronics Engineering, Beihang University, Beijing 100191, China; sheymbh@163.com (Y.S.); lantian435@163.com (T.L.); 12171038@buaa.edu.cn (X.P.); liuqianwenok@163.com (Q.L.); shangcfan@buaa.edu.cn (S.F.); 2 Science and Technology on Metrology and Calibration Laboratory, Beijing 100095, China

**Keywords:** multilayer MoS_2_ diaphragm, resonator, viscous air damping, Fabry-Perot interference

## Abstract

We demonstrated a multilayer molybdenum disulfide (MoS_2_) nanomechanical resonator by using optical Fabry-Perot (F-P) interferometric excitation and detection. The thin circular MoS_2_ nanomembrane with an approximate 8-nm thickness was transferred onto the endface of a ferrule with an inner diameter of 125 μm, which created a low finesse F-P interferometer with a cavity length of 39.92 μm. The effects of temperature and viscous air damping on resonance behavior of the resonator were investigated in the range of −10–80 °C. Along with the optomechanical behavior of the resonator in air, the measured resonance frequencies ranged from 36 kHz to 73 kHz with an extremely low inflection point at 20 °C, which conformed reasonably to those solved by previously obtained thermal expansion coefficients of MoS_2_. Further, a maximum quality (*Q*) factor of 1.35 for the resonator was observed at 0 °C due to viscous dissipation, in relation to the lower Knudsen number of 0.0025~0.0034 in the tested temperature range. Moreover, measurements of *Q* factor revealed little dependence of *Q* on resonance frequency and temperature. These measurements shed light on the mechanisms behind viscous air damping in MoS_2_, graphene, and other 2D resonators.

## 1. Introduction

Nanomechanical resonators have been employed as tools to measure force [1], mass [2], charge [3], and displacement [4] with exquisite sensitivity. Recently, two-dimensional (2D) crystals, such as graphene and molybdenum disulfide (MoS_2_), have been enabling a new class of atomically thin nanoelectromechanical systems (NEMS) for sensing and actuation functions [5] due to the vanishing of bending rigidity with decreasing thickness [6]. Among the 2D materials employed for the fabrication of these devices, graphene has drawn the most attention and has been extensively studied so far [7,8,9,10], because of its high strength, stiffness, and thermal conductivity along the basal plane. For example, Bunch et al. fabricated suspended single- and multi-layer graphene sheets over trenches and measured the fundamental frequency of 1 MHz to 170 MHz and a *Q* factor of 20 to 850 at room temperature and a pressure of <10^−6^ Torr [7]. Additionally, a suspended graphene nanoribbon resonator with all-electrical high-frequency actuation and detection presented that *Q* factor increased with decreasing temperatures, reaching ~1 × 10^4^ at 5 K [8]. Clamping graphene membranes on all sides can reduce the variation in resonance frequency and make the behavior more predictable [9]. Hence, Barton et al. measured circular graphene mechanical resonators of various diameters using optical excitation and detection, and obtained a *Q* factor as high as 2400 ± 300 for a resonator with a diameter of 22.5 μm at room temperature and a pressure of <6 × 10^−3^ Torr [10].

Similar to graphene, MoS_2_ also has a hexagonal crystal structure [11] and demonstrates the potentially optical and optoelectronic applications [12]. Recent theoretical models using classical molecular dynamics simulation have demonstrated the possibility of lower intrinsic energy dissipation in MoS_2_ [13]. Furthermore, ultralow areal mass density of 3.3 fg/μm^2^, high elastic modulus (~0.3 TPa), and an exceptional strain limit of 10%~12% [14] make it an attractive alternative to graphene, which typically has higher energy dissipation [15], especially in air damping due to a smaller thickness per unit layer and lower mass density [7]. For these reasons, much effort has been made to characterize the resonance behaviors of NEMS devices made of single-layer, few-layer, or multilayer MoS_2_ films in a vacuum chamber using electrical or optical excitation at room temperature [16,17,18,19]. Lee et al. reported on the demonstration of MoS_2_ nanodevices, where MoS_2_ diaphragms as thin as 6 nm exhibited fundamental-mode nanomechanical resonances up to ~60 MHz in the very high frequency band, and frequency-*Q* factor products up to ~2 × 10^10^ Hz [16]. Andres et al. fabricated suspended single-layer MoS_2_ resonators, which behaved with resonance frequencies in between 10 and 30 MHz and *Q* factors in between 16 and 109 [17]. Then, Kramer et al. experimentally studied the effect of mechanical strain on the dynamics of thin 15-nm-thick MoS_2_ nanodrum resonators with a diameter of 5 μm by using a piezoelectric bender to introduce strain [18]. Recently, Lee et al. further investigated γ-ray radiation effects on MoS_2_ nanodrum resonators with diameters of 5–6 μm by using optical interferometric resonance readout, which vibrates at megahertz frequencies [19].

Accordingly, these experimental studies have significantly advanced the understanding of MoS_2_ resonance behaviors. However, these approaches typically involve experiments where MoS_2_ flakes in adhesive contact with a substrate are mechanically exfoliated by well-controlled forces, and the motions of these resonators are detected using an optical interferometer in vacuum pressure [15,16,17,18,19]. In fact, air damping is an important dissipation mechanism when nanomechanical resonators are operated in a moderate vacuum or near ambient, and resonance characteristics in air are completely different from those in a vacuum chamber [6]. Moreover, a complicated sample test process and specific measurement setups, such as a beam expander, a lens, a dichroic mirror, etc., are generally needed for the free-space optical excitation and detection technique used in the aforementioned literature. Hence, in this paper, from the viewpoint of MoS_2_ device application instead of the aforementioned sample analysis, we fabricated a simple and miniature optical fiber Fabry-Perot (F-P) resonator with MoS_2_ diaphragm to study the effect of viscous air damping on the F-P resonator in consideration of temperature changes, which contributes to the sensitive structural improvement and packaging design of F-P resonators. Moreover, we reported on experimental measurement of resonance behaviors of a chemically vapor deposited (CVD) MoS_2_ diaphragm in air damping, which was suspendedly adhered to a zirconia (ZrO_2_) ferrule endface with a diameter of 125 μm, much larger than several microns in previously reported resonators [7,8,9,10,15,16,17,18,19]. Then, an F-P cavity made of the transferred MoS_2_ diaphragm and a fiber end was easily formed to offer photothermal excitation and motion detection by using a simple F-P interference scheme rather than the complicated free-space optical actuation scheme mentioned above. Finally, thermal effects in the range of −10–80 °C were tested, which showed that resonance frequencies in contrast to *Q* factors strongly depended on the temperature due to thermal strain of the nanomembrane in viscous damping regime instead of previously described intrinsic damping or free molecule flow damping [5,20].

## 2. Fabry-Perot (F-P) Resonator Fabrication and Optical Actuation Model

**Figure 1:**
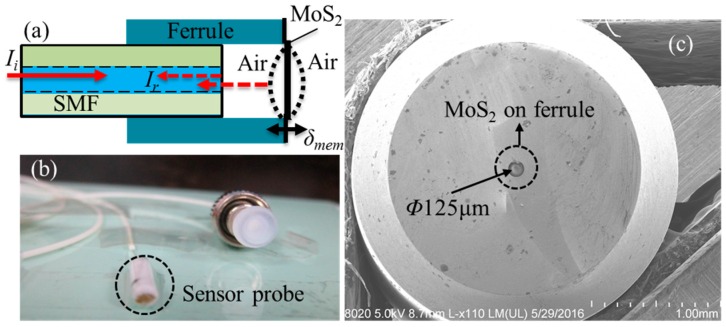
(**a**) Schematic diagram and (**b**) physical picture of the Fabry-Perot (F-P) sensor probe. (**c**) Microscopic image of the molybdenum disulfide (MoS_2_) diaphragm adhered to ferrule.

Figure 1a,b show the schematic diagram and the physical picture of the presented F-P sensor probe that is comprised of a zirconia ferrule, a standard single mode fiber (SMF), and a multilayer MoS_2_ diaphragm. The diaphragm, working as a light reflector made directly on the end of the SMF, is adhered to the zirconia substrate by van der Waals forces, and the initial cavity length of 39.92 μm between the fiber end and the ferrule endface is solved by an optical spectrum analyzer (AQ6370) to achieve the preferable reflected light intensity via a 1-μm resolution translation stage. As shown in Figure 1c, the 125-μm diameter hole is covered by the diaphragm. Then, the ferrule and the SMF are held together by an epoxy adhesive (3M^®^).

**Figure 2:**
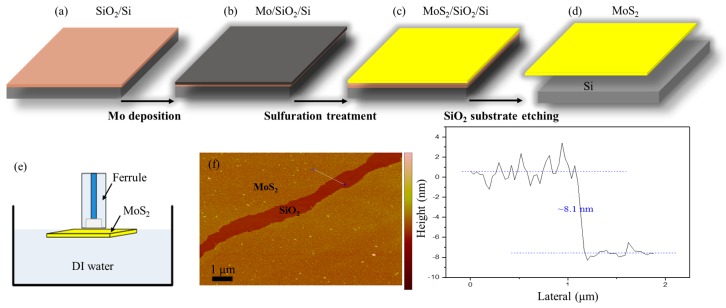
Fabrication process of the F-P cavity with a MoS_2_ membrane. (**a**) SiO_2_/Si substrate. (**b**) Depositing Mo film onto the SiO_2_/Si substrate. (**c**) Treating the Mo/SiO_2_/Si assembly with sulfurization. (**d**) Separating MoS_2_ membrane from the MoS_2_/SiO_2_/Si assembly. (**e**) MoS_2_ membrane-covered fiber-capillary tip. (**f**) Thickness measurement of MoS_2_ membrane by atomic force microscopy (AFM).

The process for preparing the multilayer MoS_2_ membrane and transferring it onto the ferrule endface is depicted in Figure 2. Referring to Figure 2a,b, an atomically thin Mo membrane is deposited onto the SiO_2_/Si substrate by an Anelva L-400 EK e-beam evaporation (Canon Anelva Corporation, Kanagawa, Japan). Then, the Mo/SiO_2_/Si assembly is loaded into a CVD system operating at atmospheric pressure for sulfuration treatment as shown in Figure 2c. Note that the CVD system is firstly rinsed with Ar to purge the quartz tube with inert atmosphere, and the quartz tube reactor is then heated up to 750 °C with a rate of ~10 °C/min under Ar atmosphere of 300 sccm. Besides, when the Mo/SiO_2_/Si Mo/Si assembly is transferred to the CVD system, it will be rinsed with ethyl alcohol and then dried with high purity nitrogen to avoid the possible surface contaminations. After the sulfurization, the MoS_2_ membrane will be grown on the SiO_2_/Si substrate, and the MoS_2_/SiO_2_/Si assembly is then put in the cold zone of the CVD reactor to make fast cooling, which contributes to reducing the crystallinity of MoS_2_ [21]. Next, the grown MoS_2_ membrane can be separated from the substrate by etching away the SiO_2_ layer by hydrofluoric acid as shown in Figure 2d. The membrane is then transferred into de-ionized (DI) water. The following step is to suspend the membrane onto the endface of a ferrule. As illustrated in Figure 2e, the ferrule is moved down slowly toward the floating MoS_2_ membrane in DI water until it touches the membrane. The membrane is then attached to the endface of the ferrule by a van der Waals interaction. The MoS_2_ membrane-covered fiber-capillary tip assembly is then left to dry in a cabinet at a room temperature, which is required to be below 50 °C for about half an hour. After water evaporation, the MoS_2_ membrane is firmly stuck to the surface of the fiber-tip to form one light reflector of a sealed F-P microcavity. The magnified microscope image of MoS_2_ diaphragm on the surface of the assembly is shown in Figure 1c. The exact thickness of MoS_2_ membrane is confirmed by atomic force microscopy (AFM). From the height profile of the MoS_2_/SiO_2_/Si assembly in Figure 2f, the thickness of the fabricated MoS_2_ membrane is determined to be ~8 nm.

According to the theory of multiple-beam interference, the interference intensity *I_r_* in the fabricated F-P cavity in Figure 1a can be expressed as [22]

(1) $$I_{r}=\frac{R_{2}+\xi R_{1}+2\sqrt{\xi R_{1}R_{2}}\cos\theta }{1+\xi R_{1}R_{2}+2\sqrt{\xi R_{1}R_{2}}\cos\theta }I_{i},$$

where *R*_1_ and *R*_2_ are, respectively, the reflectivities of MoS_2_ membrane and the fiber end face. *R*_2_ is measured to be 2.5%, and *I_i_* is the incident intensity. *ξ* is the coupling coefficient of cavity length loss [23]. When ignoring the half-wave loss, the phase difference *θ* between two adjacent beams in micro-air cavity can be written as

(2) θ=4πL/λ,

where *L* is the length of F-P cavity, and *λ* is the wavelength of incident light. When a diaphragm-type F-P interferometer made of low-reflectivity mirrors is illuminated by a monochromatic light source, the response is a periodic function similar to a two-beam interferometer. Based on the thin-film optical theory and the Fresnel’s equations for reflection and refraction [23], the reflectivity of MoS_2_ film is measured to be ~0.89%. Thus, due to the lower reflectivities of the two F-P mirrors, Equation (1) can be simplified as

(3) $$I_{r}=\left(R_{2}+\xi R_{1}+2\sqrt{\xi R_{1}R_{2}}\cos\theta \right)I_{i},$$

It should be noted that applied optical excitation will cause the suspended MoS_2_ diaphragm to vibrate due to thermal expansion and contraction. It is assumed that the displacement of diaphragm vibrating axially with simple harmonic motion is $\delta_{mem}=A\sin\left(2\pi ft+\varphi \right)$, where *A* is the amplitude, *f* is the frequency, and *φ* is the phase angle. As a result of the resonance motion of diaphragm, *I_r_* can be rewritten as

(4) $$I_{r}=\left[R_{2}+\xi R_{1}+2\sqrt{\xi R_{1}R_{2}}\cos\left(\theta +4\pi \delta_{mem}/\lambda \right)\right]I_{i},$$

when the diaphragm does not vibrate, *δ_mem_* = 0 and Equation (4) can then degenerate to Equation (3).

**Figure 3:**
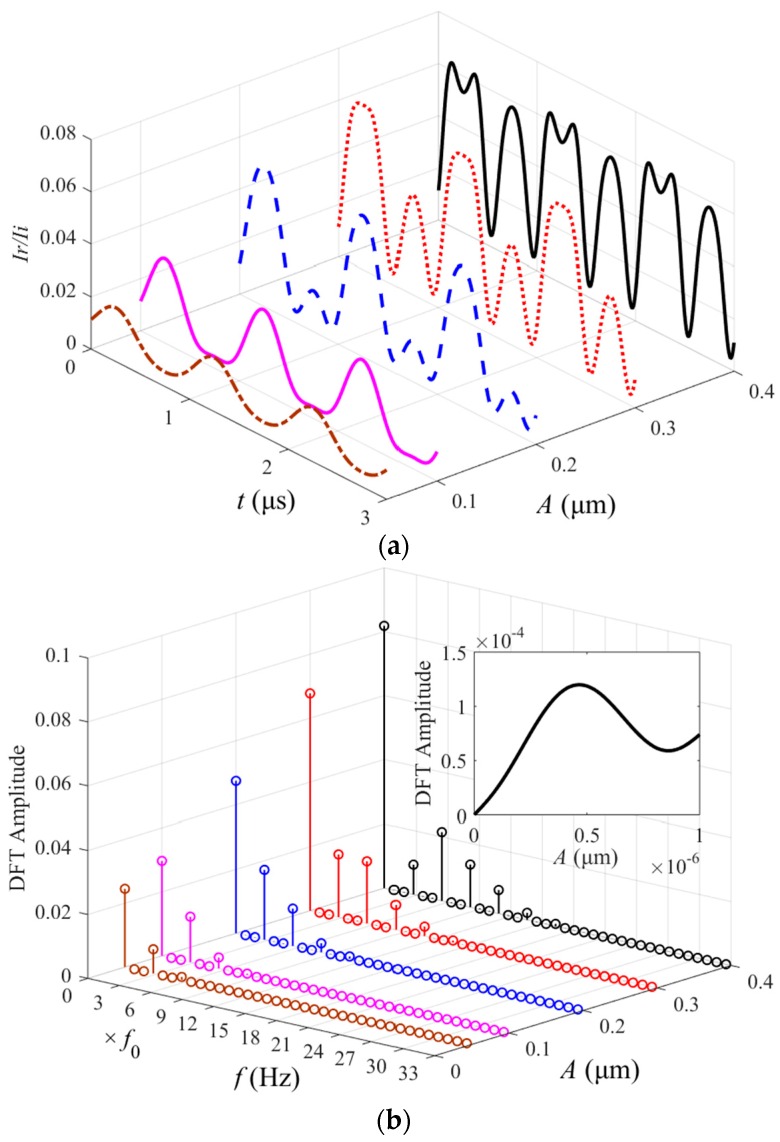
The effect of *A* on *I_r_*/*I_i_*. (**a**) Time-domain waveforms. (**b**) Frequency-domain waveforms. Inset: the discrete Fourier transform (DFT) amplitude-*A* response at *f*_0_.

As seen from Equation (4), the reflected light intensity for F-P optical readouts shows a dependence on *δ_mem_*. Thus, the determination of resonance frequencies and *Q* factors is closely related with *δ_mem_*. In other words, the resonance characteristics of MoS_2_ resonator as a light reflector can be measured by using an extrinsic F-P interferometric scheme. However, the *δ_mem_*-related incident light intensity should be adjusted to an appropriate range instead of an arbitrary higher value. Assuming the frequency of *δ_mem_* for the diaphragm is 53 kHz, which is defined as *f*_0_. It can be inferred from the simulation results in Figure 3, where *N × f*_0_ (*N* = 0, 1,…) represents the *N*-order fundamental frequency, that *I_r_*/*I_i_* exhibits more obvious waveform distortions and frequency multiplication patterns with rolling energy distribution when the amplitude *A* of *δ_mem_* is set as larger values, such as 0.1~0.4 μm. The phenomenon will result in nonlinear mode coupling and internal resonances related with complex energy transfer between various vibrational modes [15]. However, the discrete Fourier transform (DFT) amplitude of resonance response at *f*_0_ does not monotonically increase with *A* (Figure 3b, inset). In other words, merely a higher light intensity for obtaining the resonance with greater amplitude is not desirable. Therefore, appropriate excitation schemes, such as incident light intensity or signal modulation, should be considered properly for a certain material of resonator in view of its photothermal absorption property.

## 3. Experiment and Analysis

**Figure 4:**
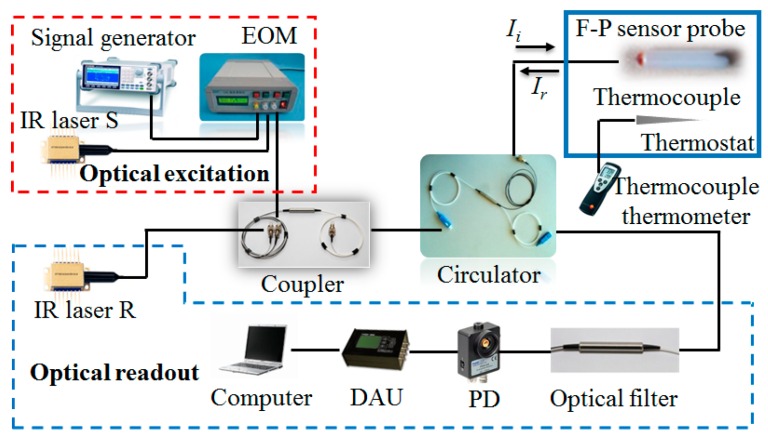
Schematic illustration of optical fiber F-P excitation and detection.

Referring to Figure 4, to detect the resonance of the MoS_2_ drum, we used an optical fiber F-P interferometric method rather than the free-space optical actuation one described previously [7,9,16,17,18,19]. Resonator motion was actuated using a 1550.12-nm amplitude-modulated distributed feedback (DFB) laser S (10 dBm output power) that excited motion through photothermal expansion and contraction of the MoS_2_ diaphragm via an electro optic modulator (EOM) with power amplification. Then, resonator motion was monitored by another 1551.72 nm DFB laser R (10 dBm output power) reflecting from the resonator and the fiber tip. The light signals from the lasers S and R were optically coupled through a 2 × 1 coupler, followed by input into the sensor probe via a 3-port circulator. The F-P interference was related with the reflected light intensity *I_r_* when the resonator vibrated. After the separation of detection light with a wavelength of 1551.72 nm by an optical filter, the intensity was detected by a fast photodiode connected to a data acquisition unit (DAU). To estimate the effects of temperature on resonance characteristics, the F-P sensor probe and a thermocouple sensor were put inside a thermostat. The reference temperature was offered by a thermocouple thermometer (testo 925) with an accuracy of ± (0.5 °C + 0.3% of measured value) in the range of −40 °C to +900 °C and an associated Type K thermocouple probe with an accuracy of Class 2 in the range of −60 °C to +400 °C according to standard EN 60584-2. Considering the use of room temperature curable adhesive and common SMF, the tested temperature range was set as −10–80 °C with an interval of 10 °C. All resonance measurements were performed at atmospheric pressure, where viscous damping was found to be significant because the Knudsen number, *K_n_ = λ_MFP_*/*l_device_*, where *λ_MFP_* is the mean free path of air molecules and *l_device_* is MoS_2_ device characteristic length, was solved as 0.0025~0.0034 in the temperature range mentioned above [5,24].

**Figure 5:**
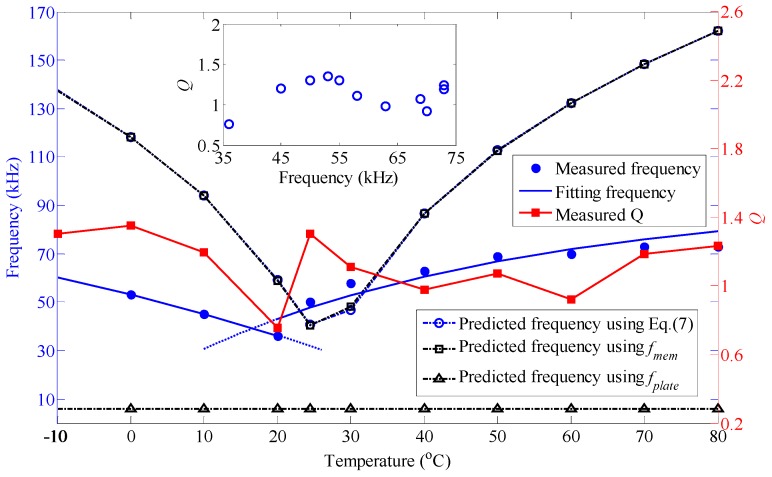
Resonance frequency *f* and *Q* factor as a function of temperature. Inset: *Q* factor verse *f*.

Since clamping the membranes on all sides made the distribution of higher resonance modes relative to the fundamental modes predictable [9], the resonance behavior of the clamped circular elastic MoS_2_ diaphragm adhered to the ferrule endface as a function of temperature was examined. From Figure 5, the measured resonance frequency scales approximately as *T^m^* where *m* = −5.348 from −10 to 20 °C, and as *T^n^* where *n* = 3.143 from 20 to 80 °C. The frequency of the resonator at 20 °C achieves an extremely low value of 36 kHz. The origin of the frequency tunability with temperature is the change in tension in MoS_2_ primarily due to the expansion/contraction of substrate and MoS_2_ similar to the thermal expansion of graphene [25]. Thus, the total elastic strain *ε*(*T*) at a given temperature *T* in MoS_2_ is approximately defined by

(5) $$\varepsilon \left(T\right)=\int_{297}^{T}\left[\alpha_{s}\left(K\right)-\alpha_{mem}\left(K\right)\right]dK,$$

where *α_mem_*(*K*) and *α_s_*(*K*) are the coefficients of thermal expansion of substrate and MoS_2_ [26], respectively. Since *ε*(*T*) is measured relative to the strain at room temperature (297 K), the change in tension in the membrane can be described as ΔS(T)=Etε(T)/(1−υ), where *E*, *t*, and *υ* are the Young’s modulus, thickness, and Poisson’s ratio of MoS_2_, respectively. According to the membrane-to-plate behavior, the resonance frequency of the resonator in a cross-over regime can be calculated as [17]

(6) $$f=\sqrt{f_{mem}^{2}+f_{plate}^{2}}=\sqrt{\frac{0.146}{a^{2}}\frac{S}{\rho t}+\frac{0.22Et^{2}}{\rho \left(1-\upsilon^{2}\right)a^{4}}},$$

where *a* is the radius of MoS_2_; *ρ* represents the mass density of MoS_2_ with certain adsorbates; *S* is the tension in the membrane at *T*, which is equal to the sum of Δ*S*(*T*) and the initial tension *S*_0_ at 297 K. Moreover, the resonance frequency shift of circular MoS_2_ diaphragm in contact with air is approximately represented by [27]

(7) $$f_{air}=f/\sqrt{1+\rho_{air}a\Gamma /\left(\rho t\right)},$$

where *ρ_air_* is the density of air, and Γ is a non-dimensionalized added virtual mass incremental factor, which is a function of mode shapes and boundary conditions. For the fundamental resonant mode *f*_0,1_, Γ = 0.746. Accordingly, resonance frequency dispersion with temperature enables the estimation of *ρ* and *S*_0_ simultaneously by fitting measured frequencies based on the established continuum model. In this way, we estimated *ρ* = 10*ρ_m_* and *S*_0_ to be 0.054 N/m for the fundamental mode, where *ρ_m_* is the mass density of pure MoS_2_. Similar to graphene resonators [9,10,25], the extra mass is attributed to resist residue and adsorbates from the fabrication and store process.

Referring to Figure 5 again, in comparison with the calculated fundamental frequencies (‘○’, blue dashed line) using Equation (7), the variation of measured frequencies with temperature is well in agreement with the predicted results; however, there is a sharp frequency deviation up to 63.47 kHz between them, which is to a great extent caused by the presence of non-uniformly distributed adsorbates [17], the geometric imperfection such as microscopic wrinkles and surface-related effects [18], and the pressure change in the F-P cavity covered with MoS_2_ membrane due to thermal fluctuations [28]. It should be pointed out that there also appears to be an extremely low inflection point in the predicted spectrum represented by the symbol “○” at 24 °C, which deviates about 4 °C from the measured inflection point. This shift of inflection point temperature possibly results from the non-uniformly distributed adsorbates and internal thermal deformation on the membrane. In addition, the resonance frequencies *f_air_* are predicted based on Equation (7) using *f_mem_*, *f_plate_*, and *f* in the membrane, plate, and cross-over regime, respectively. It is inferred from *f_air_* plotted as by the symbols ‘□’, ‘Δ’, and ‘○’ that the resonator made of multilayer MoS_2_ should behave as a circular membrane dominated by initial pre-tension and thermal strain-dependent additional tension. Then, the *Q* factor can be extracted from the full width half-maximum of the Lorentzian resonance peak; nevertheless, there is no clear dependence of *Q* on temperature. The highest *Q* factor observed is ~1.35 at 0 °C for the resonator surrounding the air in tested temperature range, i.e., lower temperatures contribute to higher *Q* factors. The inset in Figure 5 shows that *Q* factor from ~0.7 to ~1.35 fluctuates around 1, which is weakly dependent on fundamental frequencies. However, the change of *Q* factor is quite significant in the limited test frequency ranging from 35 kHz to 75 kHz, which is in contrast to top-down fabricated microelectromechanical systems (MEMS) resonators, such as silicon nitride nanostrings, where a fitted linear relationship is achieved between *Q* factors from 17 to 600 and resonant frequencies from 7 MHz to 206 MHz by tuning operating pressures at room temperature [20]. Therefore, structural size is an essential factor determining the *Q* of the membrane [29], which is similar to the behavior of graphene resonator previously reported [10]. It is important to note that the experiments on resonance behavior versus temperature were repeated three times.

**Figure 6:**
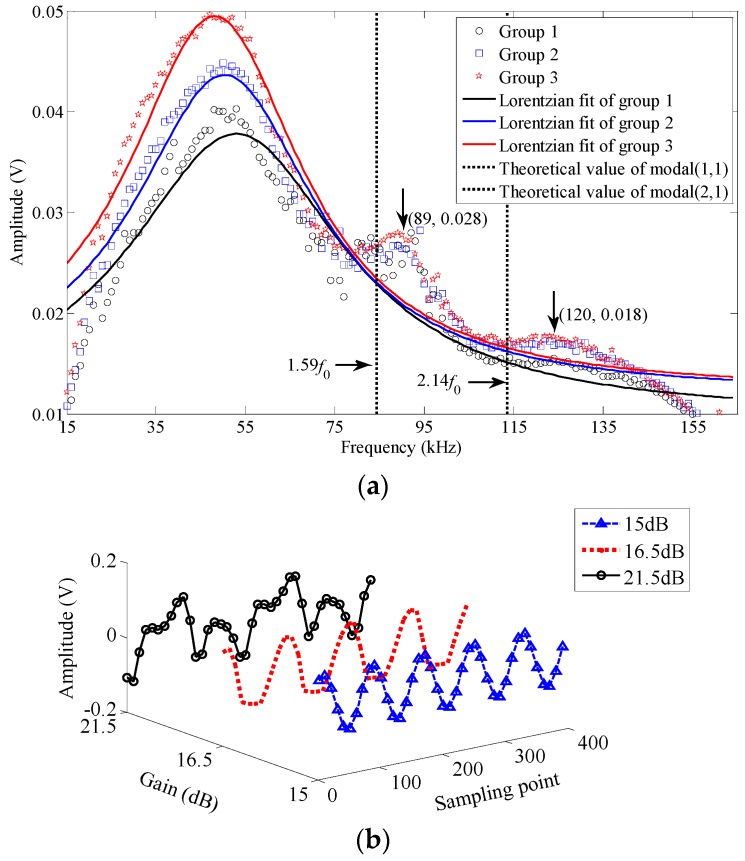
(**a**) Measured resonance spectrum and (**b**) nonlinear distortion due to increasing optical power at 0 °C.

Figure 6a illustrates the resonance spectrum with an average fundamental frequency of *f*_0_ = 53 kHz and a maximum average value of *Q* = 1.35 at 0 °C. The resonator demonstrated little deviation from a harmonic response at this temperature due to the tensioned-membrane size effect in air damping. By averaging, two higher eigenmodes at 1.68 *f*_0_ (~89 kHz) and 2.26 *f*_0_ (~120 kHz) are found, which are in reasonable agreement with the predicted higher eigenmodes for a circular membrane (1.59 *f*_0_ and 2.14 *f*_0_). The discrepancies between the measured values and the predicted values are also caused by non-uniform tensioned membrane and atmospheric adsorbates on the device. Furthermore, increasing optical power will further drive the resonator beyond the regime of harmonic oscillations as observed in Figure 6b, where the sampling frequency is set as 100 MHz. A gradually clear waveform distortion relative to a 50-kHz harmonic response is noticed when the driving power gain of EOM in Figure 4 is increased to 16.5 dB and then 21.5 dB from 15 dB, in agreement with the simulated responses shown in Figure 3b. This phenomenon features the nonlinear oscillation mode coupling effect along with rolling frequency multiplication components, which arises from the alternating tension in MoS_2_ membrane due to higher vibration amplitude comparable to the membrane thickness, consistent with those recently reported for single-layer and multilayer MoS_2_ resonators in [15,17].

To sum up, the experimental results demonstrate that a simple and miniature optical fiber F-P resonator with MoS_2_ or other 2D diaphragms can be successfully fabricated and tested instead of using a complicated free-space optical actuation scheme. However, when the resonator operates in air, its *Q* factor will be significantly reduced by viscous damping dissipation. Although a lower *Q* factor is weakly dependent on fundamental frequencies in the tested frequency range, its fluctuating change is quite significant with respect to the narrow bandwidth of about 40 kHz. It should be pointed out that the resonant frequency does not exhibit monotonic change with the temperature. In fact, there appears to be an extremely low inflection point in the predicted spectrum due to internal thermal deformation on the membrane. The measured resonant frequencies differ from theoretical ones primarily because of the presence of non-uniformly distributed adsorbates, absorbed on the MoS_2_ membranes in air. Unfortunately, the adsorbates result in additional masses and tensions, which will even cause the dispersion of resonant responses for different samples. Hence, the damping mechanism-dependent Knudsen number should be regulated optimally with the trade-off of sensitive structures of F-P resonators and testing conditions including temperature and pressure. In this case, the experimental results explain the low *Q*-factors of 2D nanomechanical resonators due to viscous air damping, which contributes to the sensitive structural optimization and packaging design of F-P resonators. Further research on the sensitive structural improvement of F-P resonators is needed to reduce air damping, in consideration of membrane size effect and optical fiber interference actuation.

## 4. Conclusions

This study demonstrated a new type of mechanical F-P resonator based on a 125-μm diameter nanothick suspended MoS_2_ diaphragm using optical interference excitation and detection. Air damping effects on resonance behavior were experimentally investigated in the temperatures ranging from –10 °C to 80 °C. Due to the interaction with the air, the MoS_2_ device exhibited noticeable resonances with a maximum *Q* factor of ~1.35 at 0 °C. Based on the derived viscous damping analytical model concerned with the Knudsen number, we identified the temperature-dependent modal frequencies from 36 kHz to 73 kHz, whose variation was described well with the result obtained by theoretical thermal expansion coefficients of MoS_2_ reported in the previous study. By examining higher order modes, a qualitative description of measured nonlinear resonances was proposed through the dependence of photothermal excitation in its temperature. The experimental results shed light on the resonance behavior observed in 2D nanomechanical resonators in viscous air damping, which also contributes to the resonating performance optimization of F-P resonators with 2D diaphragm.
